# Evaluation of the Suitability of Poly(Lactide)/Poly(Butylene-Adipate-*co*-Terephthalate) Blown Films for Chilled and Frozen Food Packaging Applications

**DOI:** 10.3390/polym12040804

**Published:** 2020-04-03

**Authors:** Arianna Pietrosanto, Paola Scarfato, Luciano Di Maio, Maria Rossella Nobile, Loredana Incarnato

**Affiliations:** Department of Industrial Engineering, University of Salerno, Via Giovanni Paolo II, 132, 84084 Fisciano (SA), Italy; arpietrosanto@unisa.it (A.P.); ldimaio@unisa.it (L.D.M.); mrnobile@unisa.it (M.R.N.); lincarnato@unisa.it (L.I.)

**Keywords:** biopolymers, PLA/PBAT, blown films, food packaging, film for frozen food

## Abstract

The use of biopolymers can reduce the environmental impact generated by plastic materials. Among biopolymers, blends made of poly(lactide) (PLA) and poly(butylene-adipate-co-terephthalate) (PBAT) prove to have adequate performances for food packaging applications. Therefore, the present work deals with the production and the characterization of blown films based on PLA and PBAT blends in a wide range of compositions, in order to evaluate their suitability as chilled and frozen food packaging materials, thus extending their range of applications. The blends were fully characterized: they showed the typical two-phase structure, with a morphology varying from fibrillar to globular in accordance with their viscosity ratio. The increase of PBAT content in the blends led to a decrease of the barrier properties to oxygen and water vapor, and to an increase of the toughness of the films. The mechanical properties of the most ductile blends were also evaluated at 4 °C and −25 °C. The decrease in temperature caused an increase of the stiffness and a decrease of the ductility of the films to a different extent, depending upon the blend composition. The blend with 40% of PLA revealed to be a good candidate for chilled food packaging applications, while the blend with a PLA content of 20% revealed to be the best composition as frozen food packaging material.

## 1. Introduction

In recent years, the growing interest of the population towards environmental issues, and the increasingly stringent directives that various countries are adopting to fight against environmental pollution deriving from plastic materials, have directed research towards the development of ecofriendly materials that can substitute the conventional plastic ones [1]. The packaging sector is the one that uses the highest amount of plastic [2], and thus, the use of so-called “biopolymers” in the food packaging field could be a strategy to reduce the environmental impact generated by the traditional plastics materials [3]. Moreover, since a considerable amount of foods requires sub-ambient storage temperatures (e.g., chilled and frozen food), the retention of satisfactory performance properties at low temperatures has a key role to extending the range of applications of a material in the food packaging field.

Different examples can be found in literature on the effect of sub-ambient temperature upon the mechanical and barrier response of conventional polyolefin-based systems [4,5,6,7], since, currently, these are the most employed materials for low temperature packaging applications.

As a general trend, in terms of mechanical response, the temperature lowering gives a decrease in the material ductility and shock absorption power; instead, in terms of barrier properties, it gives a decrease of permeability to gases and a change in perm-selectivity. To our best knowledge, no literature data are available on the effect of sub-ambient temperatures on the functional performances of biopolymer-based packaging systems, and their applications for refrigerated food storage are still very limited.

Among the biopolymers, PLA is one of the most promising and widespread ones that can replace petrochemical plastics [8,9,10]. PLA is a compostable and bio-based polymer, it is a linear aliphatic polyester, and its properties can be turned varying the relative content of D- and L-enantiomers [11]. It can be easily processed with conventional process such as extrusion, spinning, injection and compression molding [12,13,14,15]. However, its high brittleness limits its use, thus different strategies are tested in order to improve its toughness [16,17,18,19]. Among them, blending of PLA with PBAT has shown promising results.

PBAT is a compostable aliphatic aromatic co-polyester that consists of two types of comonomer, a rigid butylene terephthalate segment made of 1.4 butanediol and terephthalic acid monomers, and a flexible butylene adipate section made of 1.4 butanediol and adipic acid monomers [20]. This polymer is flexible and tough, and it has complementary properties to PLA [21].

Several researchers have blended PLA with PBAT, through several techniques, like single-screw extrusion, twin-screw extrusion and solvent casting [20,22,23,24,25,26]. Results showed that, even if these polymers have very close solubility values [27], they are not thermodynamically miscible.

Li et al. [28] studied the morphology of this system in a wide range of compositions. They showed that for a PBAT content lower than 20 wt%, the blend surface appeared uniform, characterized by the presence of featured small droplets suspended in the continuous phase. When the PBAT content increased between 20 and 50 wt%, the system became heterogeneous, with a co-continuous phase structure formed at 50 wt%. Furthermore, when the PBAT content exceeded 70 wt%, the morphology turned into droplets. In addition, Deng at al. [29] investigated the morphology of these systems, and they found a fibrillar morphology with a co-continuous phase structure when the PBAT content was between 20 wt% and 40 wt%, and PLA dispersed particles in the PBAT matrix when the content of the latter polymer was between 60 wt% and 80 wt%.

However, even if these polymers are not miscible, their blending allows us to obtain several advantages. Hongdilokkul et al. [30] reported that the addition of 20 wt% of PBAT into the PLA matrix considerably improved the processability for film blowing by increasing the melt strength of the system. Moreover, the presence of PBAT influenced the crystallization behavior of PLA; in fact, it is reported that PBAT can act as a nucleating agent for PLA [30,31], increasing its crystallization rate.

Furthermore, several works deal with the mechanical properties of this system. Wang et al. [32] have melt blended PLA and PBAT, and showed that the brittleness and ductility of PLA could be improved by adding 10% PBAT, which increased the elongation at break from 7.71% to 357.8%, and decreased the tensile strength from 65.21 MPa to 47.52 MPa. Li et al. [33] have analyzed a wide range of the composition of PLA/PBAT blends, showing an increase of the elongation at break with the PBAT content in the blend, and a reduction of the elastic modulus that, in the blend with the 20% of PBAT, was decreased of more than 30% compared to the pure PLA. Farsetti et al. [34] made an impact analysis, and they reported an increase of the GIC (critical release rate of strain energy) value at compositions ranging from neat PLA down to PLA mass fraction equal to 0.8, due to a toughening effect of PBAT. In general, the addition of PBAT lead to an increase of the ductility, but to a decrease of the elastic modulus in the tensile strength of the blends.

Moreover, interesting results of these blends are reported also for the food packaging field. Tabasi et al. [35] reported that the sealability properties of PLA/PBAT blends can be tailored by varying the blend composition and PBAT crystallinity degree. Higher content of PLA in the blend and the lower crystallinity of PBAT provide lower hot-tack initiation temperature.

Li et al. [33] investigated the oxygen permeability of these blends in a wide range of compositions, and with the addition of the 0.15 wt% of a compatibilizer, they found that the oxygen permeability of PBAT was two times higher than PLA, and the permeability of the blends increased as the content of PBAT in the blend increased. Wang et al. [23] reported that the presence of PBAT can considerably improve the UV-screening properties compared to neat PLA and PLA/PBAT films, which proved to be effective in preventing the greening of fresh potatoes. Moreover, the high-water vapor permeability of these films can be useful to prevent fogging on the surface of packaging film, as revealed for fresh, green onions.

However, there is a lack of literature on the effect of the temperature on the mechanical properties of these systems. Only Gigante et al. [36] studied the effect of temperature on the fracture behavior of different PLA/PBAT blends, in which PLA was the matrix phase. By the estimation of the ductile-to-brittle transition temperature (DBTT), they highlighted that the ductile behavior for these blends appeared at temperatures higher than the PBAT glass transition temperature, and particularly, for the blends with a PBAT content between 15 wt% and 25 wt%, this happened around −23 °C.

Moreover, the decrease of the ductility is a problem particularly relevant for materials with low thickness, so for the flexible packaging. Thus, the knowledge of the mechanical behavior at low temperatures is essential information to evaluate the suitability for packaging foods requiring low storage temperatures.

Therefore, in this work, PLA/PBAT blown films in a wide range of compositions were developed and fully characterized, and the mechanical properties of the most ductile films were evaluated at three different temperatures (25 °C, 4 °C and −25 °C), in order to extend the range of application of these blends for chilled and/or frozen food flexible packaging.

## 2. Experimental

### 2.1. Materials

PLA 4032D, supplied by NatureWorks^TM^ (Minnetonka, MN, USA), has a content of D-isomer equal to 1.5 wt%, a specific gravity of 1.24 g/cm^3^ and a melting temperature between 155–170 °C. Ecoworld PBAT 009 was manufactured by Jin Hui Zhaolong (Lüliang, China); it is composed by the 29% of adipic acid, 26% of terephthalic acid and 45% of 1,4-butanediol, it has a density of 1.26 g/cm^3^ and a melting temperature around 110–120 °C. Both materials comply with USA Food and Drug Administration (FDA) and European Union (EU) regulations for direct food contact. They also conform the standards of compostability under controlled composting conditions, i.e., standards EN13432 and ASTM D 6400.

### 2.2. Preparation of the Films

PLA and PBAT pellets were dried under vacuum at 70 °C for 20 h prior to processing. PLA and PBAT at different PLA/PBAT concentrations (100/0, 80/20, 60/40, 40/60 20/80 0/100 by weight) were mixed in a Collin ZK25 co-rotating twin extruder (D = 25 mm, L/D = 42), with a screw speed equal to 100 rpm, a temperature profile ranging from 140 °C to 190 °C, and a mass flow ranging from 50 to 54 g/min. Then, the materials were cooled in a water bath. After, pure materials and each blend were dried under vacuum at 70 °C for 20 h before processing. The blown films were prepared in a multilayer plant, using two single screw extruders GIMAC (D = 12 mm, L/D = 24), with a screw speed equal to 50 rpm and a take up speed of 1 m/min. The temperature profile ranged from 190 °C to 180 °C for pure PLA, from 180 °C to 135 °C for the blends and from 160 °C to 125 °C for pure PBAT. Films were produced with a blow-up ratio (BUR) and a take-up ratio (TUR) equal to 2.5 and with constant thickness of 75 ± 5 µm.

### 2.3. Rheological Characterization

The rheological properties in the oscillatory mode of pellets of PLA, PBAT and their blends were evaluated using an oscillatory shear strain-controlled rheometer, ARES. Samples were dried under vacuum at 70 °C for 16 h prior to testing. Tests were performed with a parallel-plate geometry (d = 25 mm) with a gap of 1 mm at 180 °C under a nitrogen atmosphere, in order to minimize thermo-oxidative degradation. A strain sweep test was initially conducted to guarantee the linear viscoelastic regime for each formulation. Frequency sweep tests were conducted in triplicate, with a standard deviation less than 2%.

### 2.4. Differential Scanning Calorimetry (DSC)

Thermal analysis on the pellets of pure materials and their blends and on the produced films was performed using a Differential Scanning Calorimeter (DSC mod. 822, Mettler Toledo, Columbus, OH, USA) under a nitrogen gas flow (100 mL/min), in order to minimize thermo-oxidative degradation phenomena. The temperature programming consisted of three steps: The samples were heated from −70 to 200 °C with a speed of 10 °C/min, and held at 200 °C for 5 min. They were then cooled at −70 at 10 °C/min, and reheated to 200 °C at 10 °C/min. The crystallinity degrees, Xc, were calculated according to the following formula:

(1) $$Xc=\frac{\Delta Hm-\Delta Hcc}{\Delta Hm^{0}\times \varphi i}\times 100$$

where Δ*H*m and Δ*H*cc (J/g) are the respective heat of melting and heat of cold crystallization of PBAT and PLA, the Δ*H*m^0^ is equal to 93.6 J/g for PLA [37] and 114 for PBAT [38], and φi is the relative weight fraction of PLA or PBAT in the blends.

### 2.5. Fourier Transformation Infrared Spectroscopy (FT-IR)

Infrared spectra of the film of pure materials and their blends were obtained with a Thermo Nicolet NEXUS 600 spectrometer in attenuated total reflectance (ATR) mode, with a diamond crystal collecting 64 scans. Each spectrum was obtained within the range of 4000–400 cm^−1^ with the wavelength resolution of 4 cm^−1^.

### 2.6. Morphological Characterization

Film samples were cryo-fractured and then coated with a thin gold layer (Agar Auto Sputter Coater mod. 108 A, Stansted, UK) at 30 mA for 160 s to improve their conductivity. Afterwards, their cross sections parallel to the transversal direction (TD) were scanned by field emission scanning electron microscope (FESEM) (LEO 1525 model, Carl Zeiss SMT AG, Oberkochen, Germany). Length evaluation was performed through the SigmaScan Pro™ Software.

### 2.7. Oxygen Transmission Rate (OTR)

OTR measurements were carried out through a permeabilimeter (GDP—C 165 of Brugger), with a manometric operation, connected to a thermo-controlled bath (ThermoHaake). Before testing, the evacuation of the upper and the bottom half-cell was performed to drive away humidity and residual gases. The test temperature was set at 23 °C, and the oxygen flow to 80 mL/min, according to ISO 15105-1. The area of the tested films was 9 cm^2^. The values of the permeability coefficients (P O_2_) were obtained by multiplying the measured value of OTR by the respective thicknesses (mm) of the films.

### 2.8. Water Vapor Transmission Rate (WVTR)

Water vapor permeability tests were performed through a Water Vapor Permeation Analyzer (Model 7002—Systech Illinois, Princeton, NJ, USA), which provides a modular system for the determination of water vapor permeability using a sensor based upon P_2_O_5_. Tests were carried out according to ASTM F 1249-90 standard (with the only exception of sensor technology) at 23 °C and 50% of relative humidity. The area of the tested films was 5 cm^2^. The values of the permeability coefficients (P H_2_O) were obtained by multiplying the measured value of WVTR by the respectively thickness (mm) of the films.

### 2.9. Mechanical Properties

Tensile testing of blown films was performed on a SANS dynamometer (Sans Testing Machine Co. Ltd., Shenzhen, China) equipped with a 100 N load cell. The rectangular shape specimens (width = 12.7 mm and length = 30 mm) were extended at a crosshead speed set according to ASTM D822 standard. Mechanical properties were evaluated in the machine direction (MD).

Tests were carried out at ambient temperature (25 °C) and at controlled environment temperatures (4 °C and −25 °C), using an Environmental chamber connected to a WK650 High Precision Temperature controller and to a Liquid nitrogen container. All of the data are the average of at least seven measurements.

## 3. Results and Discussion

### 3.1. Rheological Characterization of the Pellet

**Figure 1:**
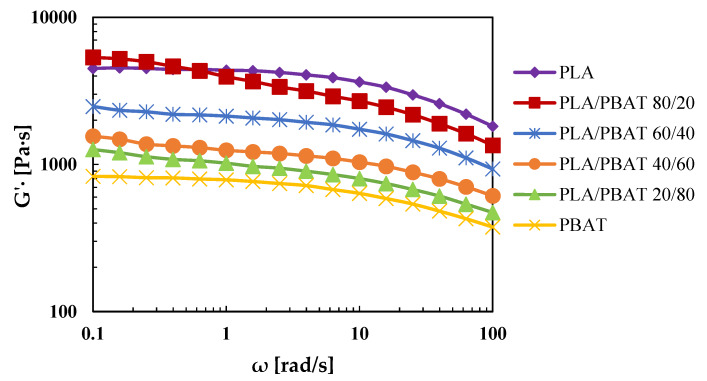
Complex viscosity of the extruded pellets.

Rheological oscillatory analysis was carried out to investigate the morphology and processability of the blends. First, a dynamic strain sweep test was performed for both the pure materials at a frequency of 10 rad/s, with the aim to determine the limit of linear viscoelasticity that was found to be greater than 5% of deformation. Thus, all of the rheological tests were performed at a strain equal to 5%. Dynamic time sweep tests were then performed to investigate the thermal stability during the flow in an inert atmosphere at the rate of 1 rad/s. These tests revealed a percentage of complex viscosity reduction in a time of 7 min (duration of a frequency sweep test) of less than 1% for both materials. Frequency sweep tests were then performed with a frequency ranging from 0.1 to 100 rad/s. The trend of the complex viscosity versus the frequency is reported in the Figure 1.

Results show that both the pure materials exhibit shear thinning behavior, and a low-frequency Newtonian plateau. Furthermore, it is evident that the complex viscosity of the PLA is higher than that of PBAT for all of the frequency tested, particularly at the viscosity ratio of 100 rad/s, when PBAT is the dispersed phase, and is equal to 0.25, and when PBAT is the matrix phase, and is equal to 4. It is well known that the viscosity ratio has a great influence on the morphology of the blends [39]. In fact, for a viscosity ratio lower than one, the application of high shear determines the formation of the fibrillar morphology of the dispersed phase. Instead, for viscosity ratio higher than one, the dispersed phase has a moderate deformation and keeps its spherical shape [40]. So, a fibrillar morphology is expected when PLA is the matrix phase, and a globular morphology is expected when PBAT is the matrix phase.

Moreover, the complex viscosity graph shows that the blends had a shear thinning behavior more accentuated than the pure materials, and the increase of PLA content led to an increase of their complex viscosity. Particularly, the PLA/PBAT 80/20 blend not only exhibited a higher viscosity, but, at low frequencies, that viscosity was higher than both PLA and PBAT, suggesting higher interactions of the two polymers at this composition [28].

**Figure 2:**
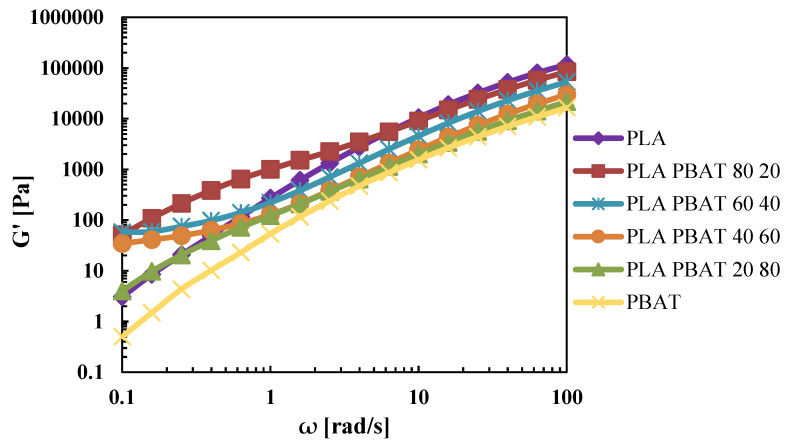
G’ of the extruded pellets.

Figure 2 shows the trend of the storage modulus (G’) in function of the frequency. The storage modulus of the blends, for high frequencies values, increased as the content of PLA in the blend increased, and instead, at low frequencies, it is higher for all the blends than the pure materials.

For PLA/PBAT 80/20 and 20/80, G’ shows a typical shoulder that has been reported for many immiscible blends [28]. This behavior of G’ is due to the “additional” elastic response originating from the surface tension of the dispersed phase in the continuous matrix. Moreover, a large G’ plateau at low frequencies is seen for the blends PLA/PBAT 60/40 and 40/60, reflecting a sort of solid-like behavior. This behavior has been reported in many investigations [41] for immiscible blends that form co-continuous structures, and this is due to extra stresses associated with the shape relaxation of the inter-connective structures [42]. In fact, as reported by Omonov et al. [43], when a continuous network structure is formed, the relaxation times shift to very low frequency values that cannot be observed experimentally, thus they used this criterion to set the composition limits of a co-continuous structure. Therefore, the 40 wt% and the 60 wt% of PBAT can be the beginning and the ending points of the co-continuous structure, which are in accordance with the values found in literature that vary from 20 wt% to 60 wt% [28,29,44].

### 3.2. Thermal Analysis (DSC) of the Films

All the films were subjected to the same thermal cycle described in the experimental part. The main thermal parameters related to the first heating are shown in the Table 1.

**Figure 3:**
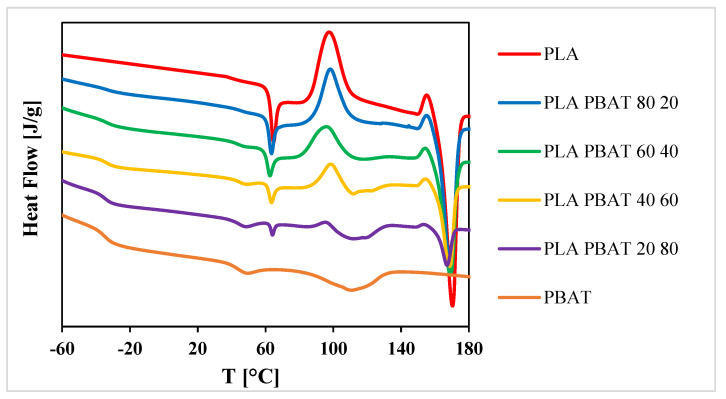
DSC thermograms of the 1st heating scan of the films.

The thermograms of the films of the 1st heating scan are reported in Figure 3.

Thermal properties have a great influence on the mechanical behavior of the films. Particularly, the glass transition temperature (T_g_) represents the transition point between the glassy and brittle behavior and the ductile behavior [45]. The glass transition temperature of PLA and PBAT are at 63 °C and −34 °C, respectively. This means that PLA has a brittle behavior at ambient temperatures (around 25 °C), and also for lower temperatures, while PBAT keeps its ductile behavior for temperatures above −34 °C. Moreover, the thermograms of the first heating of these materials show the presence of one endothermic peak for PLA at 171 °C, and two endothermic peaks for PBAT, at the temperatures of 36 °C and 107 °C.

The first melting peak, not very intense, can be attributed to the melting of the crystalline phase of the butylene-adipate (BA) fraction [46], while the second melting peak, more intense and widened, is between 70 °C and 140 °C. All the blends showed two different glass transition temperatures, and this is a further confirmation that PLA and PBAT are not thermodynamically miscible [47]. The presence of PBAT led to a decrease of the cold crystallization temperature (T_cc_) of PLA, indicating that PBAT increases the crystallization rate of PLA, as previously reported [33,48]. Moreover, as reported by Deng et al. [29], the crystallinity degree (X_c_) of PLA increases with the increase of the PBAT content in the blend. This behavior could be due to the greater mobility of the PLA chains in the presence of the PBAT. On the other hand, the opposite behavior is shown for the degree of crystallinity of PBAT, which decreases as the PLA content in the blend increases. Since PLA has a lower chain flexibility and higher viscosity than PBAT, it restricts the mobility of the PBAT chains, and therefore reduces its crystallinity degree [20].

### 3.3. FT-IR

**Figure 4:**
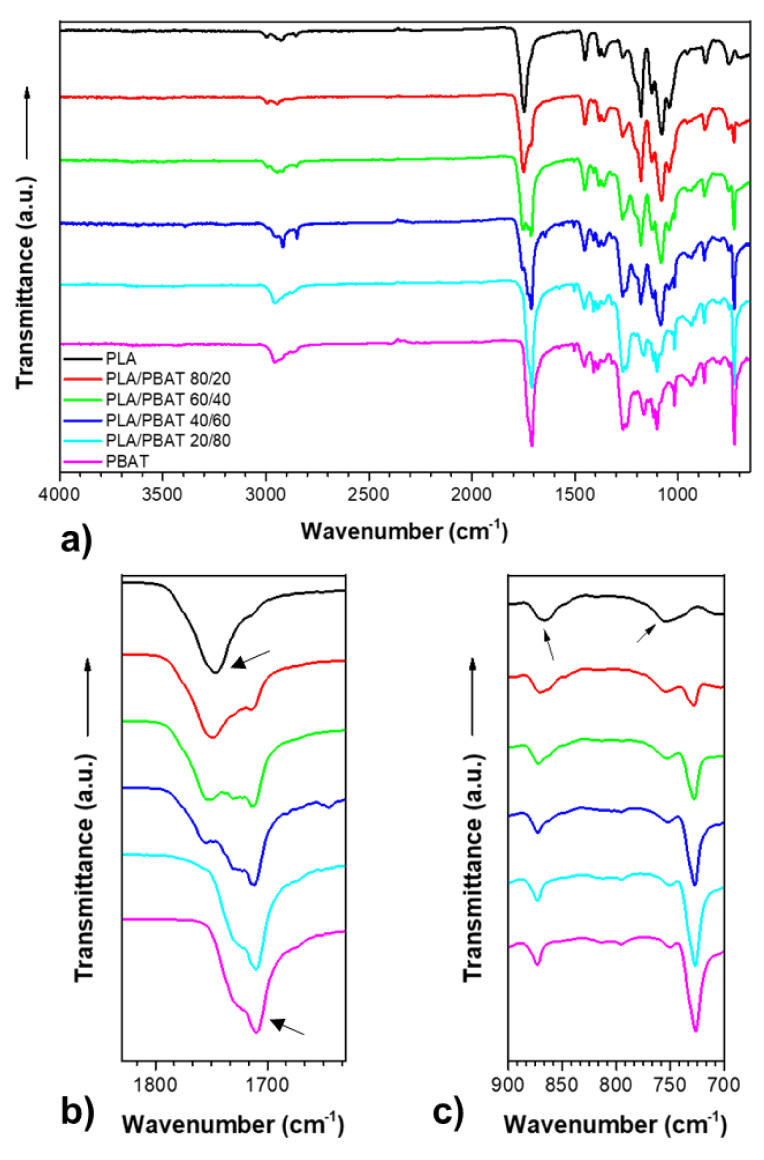
Fourier transform infrared spectroscopy (FTIR) spectra of the films: (**a**) in the full x-axis scale (**b**) zoom of the carbonyl bonds (**c**) zoom of the C–OCC and CO bonds.

FTIR spectroscopy investigations were performed on the films in order to obtain information on blends morphology, and the results are reported in the Figure 4.

According to Al-Itry et al. [49], the following main characteristic peaks for PLA can be observed: The symmetric stretching vibration of the axial CH groups in saturated hydrocarbons at 2800–3000 cm^−1^, the intense peak originating from the C=O stretching vibrations located at around 1747 cm^−1^, a weaker band at 1250–1050 cm^−1^ as a result of the C–O carboxyl groups, and two weak peaks at 866.5 cm^−1^ and 75,464 cm^−1^ as a result of the C–OCC bond stretching and CO bending, respectively. The functional groups of PBAT can be described as: Peaks at around 3000 cm^−1^ representing C–H stretching in aliphatic and aromatic portions; at around 1710 cm^−1^ representing carbonyl groups (C=O) in the ester linkage; at 1267 cm^−1^ representing C–O in the ester linkage; and a sharp peak at 720 cm^−1^ representing four or more adjacent methylene (–CH2–) groups. Bending peaks of the benzene substitutes are located at wave numbers between 700 and 900 cm^−1^. In the blends, some peak shifts were detected: The peak of the PLA carbonyl group shifts as the PBAT content increased from 1747 cm^−1^ to 1753 cm^−1^ in the PLA/PBAT 60/40 blend; the peak of the carbonyl group of PBAT moved as the PLA increases from 1710 cm^−1^ to 1714 cm^−1^ in the PLA/PBAT 60/40 blend; the peak at 866.5 cm^−1^ of PLA C-OCC moved progressively to values between 870 and 872 cm^−1^ as the content of PBAT in the blend increased, and the peak relative to the CO bending moved up to 750 cm^−1^ in the blend PLA/PBAT 20/80. These shifts indicate that these polymers, even though they are immiscible, have some interactions, and a certain degree of compatibility.

### 3.4. Morphological Characterization

**Figure 5:**
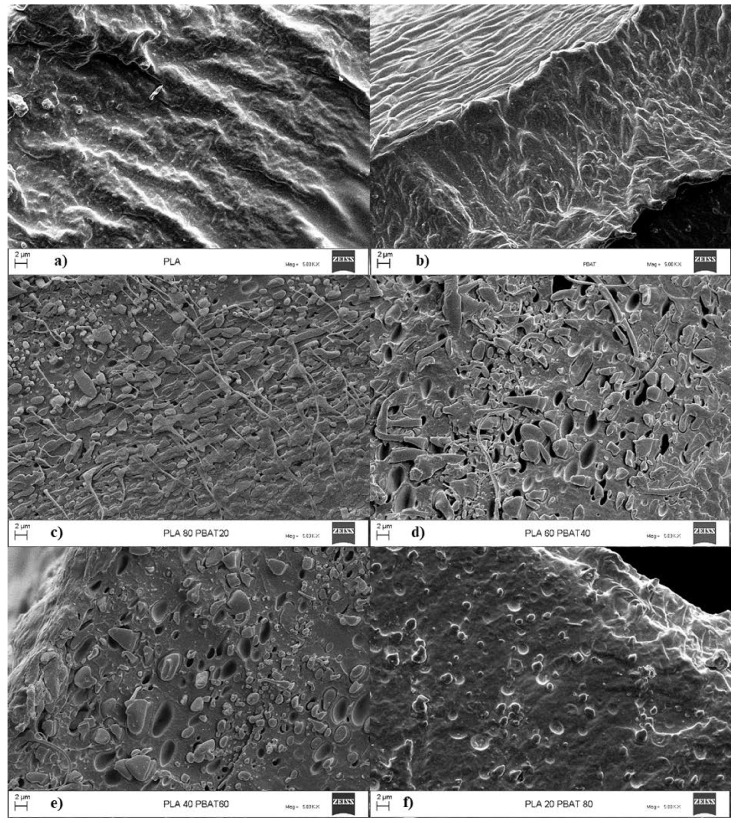
Field emission scanning electron microscope (FESEM) images of: (**a**) neat poly(lactide) (PLA), (**b**) neat poly(butylene-adipate-co-terephthalate) (PBAT), (**c**) PLA PBAT 80/20, (**d**) PLA PBAT 60/40 (**e**) PLA PBAT 40/60, (**f**) PLA PBAT 20/80.

Figure 5 gives the FESEM images of the fracture surface of the films for the neat polymers and their blends. PLA and PBAT show homogeneous fracture surfaces. On the contrary, all the blends exhibit a typical two-phase structure characterized by the presence of voids and inclusions of variable shape and dimension. Different morphologies of the dispersed phase can be observed for these blends. When PBAT was the dispersed phase, the blends showed an elongated fibrous morphology, yet instead when PBAT became the matrix phase, the morphology turned into globular, in accordance with their viscosity ratios. Particularly, the blend with the 80% of PLA showed an elongated dispersed phase with thin fibrils that confirms the higher interactions of the two polymers in this composition supposed in the rheological analysis. Moreover, the blends with a content of the dispersed phase equal to 40% showed bigger inclusions than the other blends.

In fact, higher concentrations of the dispersed phase can lead to higher coalescence, and so to bigger dimensions of the aggregates [44]. However, discrete domains that are not part of a network structure can be observed for both the blends with a dispersed phase content of 40 wt%, suggesting that the “fully co-continuous structure” could be formed in a composition between this range.

### 3.5. Barrier Properties

Oxygen and moisture barrier properties are among the most important issues to be considered in materials intended to be used in food packaging, since their presence in some cases may lead to detrimental changes in quality that result in a decrease of the food shelf-life. In Table 2 the permeability coefficient to oxygen and water vapor are reported.

From the reported table it is clear that PBAT showed significant lower barrier properties to both oxygen and water vapor than neat PLA; in fact, from the above-reported results, it is clear that adding PBAT to PLA results in an increase of the coefficient of permeability, as reported in literature [23,33]. This could be related to the different glass transition temperatures of these polymers. PLA at 23 °C is in the glassy state, which means a lower fraction of free volume between the polymer chains, and a reduced mobility compared to PBAT that has a Tg under 23 °C. These factors greatly influence the permeability of gas molecules, whose permeability is facilitated with high chains mobility and free volume fractions.

Moreover, for polymer blends, it is useful to predict the macroscopic properties, thus different models were applied in order to find the best fitting for the oxygen permeability of PLA/PBAT blends. In the Maxwell equations, the two different polymers are replaced with two bars consisting of a rubbery and a glassy phase.

In the parallel model, the two bars are connected in parallel, and in the series model they are connected in series. These models represent the highest and lowest limits for the permeability coefficient of a polymeric blend. The Maxwell series and parallel model [49,50] are respectively:

P_b_ = P_1_ · P_2_/(φ_1_ · P_2_ + φ_2_ · P_1_)

P_b_ = φ_1_ · P_1_ + φ_2_ · P_2_

where Pb is the permeability of the blend, P_1_, and P_2_ are the permeabilities of the respective phases, with φ_1_ and φ_2_, being the corresponding volume fractions.

Robeson’s equation describes the effect of a spherical filler on the overall composite permeability [51]. The equation is reported below:

Pb = Pm · [Pd + 2Pm − 2 · φd · (Pm − Pd)]/[Pd + 2Pm + φd · (Pm − Pd)]

where Pb is the permeability of the blend, Pm is the permeability of the matrix, Pd is the permeability of the disperse phase, and φd is the volume fraction of dispersed phase.

**Figure 6:**
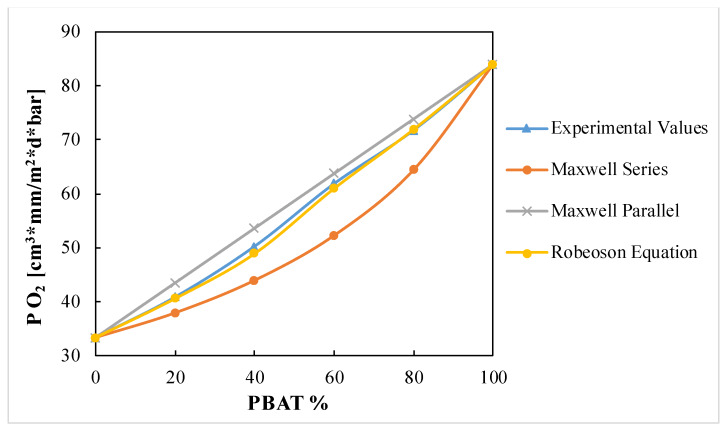
Oxygen Permeability in function of the blends’ composition.

Figure 6 shows that the experimental values of oxygen permeability are between the Maxwell series and the parallel equations. The curve that fits best the experimental results is the Robeson equation, particularly for the PLA/PBAT 80/20 and 20/80 samples, likely due to dispersed morphology of these compositions. Thus, the Robeson equation could be applied for the prediction of the oxygen permeability coefficient of the PLA/PBAT blends.

### 3.6. Mechanical Properties

**Figure 7:**
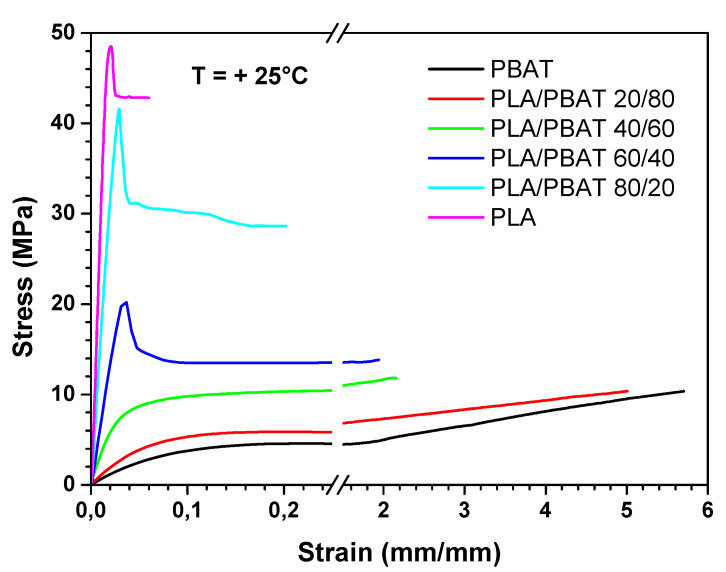
Tensile stress–strain curves, obtained at 25 °C, for all the films.

Results of the tensile tests performed at ambient temperature are reported in Figure 7 and in Table 3.

From Figure 7, it is evident that PLA and PBAT have an opposite behavior: the first is rigid and brittle, and the second is flexible and tough. Moreover, the blend films exhibit behavior intermediate between that of the neat components, showing a progressive change from fragility to ductility as the PBAT content grows.

In particular, with respect to the neat PLA film, the blend at the highest PBAT loading has a decrease of the elastic modulus (E) and yield stress (σy) of an order of magnitude, and an increase of the elongation at break (εb) of two orders of magnitude.

Similar trends in the mechanical behavior of PLA/PBAT blends are reported in the literature also by others [32,33]. On the contrary, Deng et al. [29], reporting on a series of melt-blended PLA/PBAT, found a drop of the elongation at break for a PBAT mass content between 50% and 60%.

Moreover, as for the oxygen permeability, it can be useful to predict the mechanical properties of a polymeric blend. Particularly, the elastic modulus is strongly dependent on the blend morphology and composition, and could be useful to assess the miscibility in polymers blends [52]. The Parallel and the Series Model [53] represent the highest and the lowest bound of the modulus of a polymer system, respectively:

E_b_ = E_1_φ_1_ + E_2_ φ_2_ (2)

1/E_b_ = 1/(E_1_φ_1_ + E_2_ φ_2_) (3)

Another model is the Davies Equation [54], which describes the elastic modulus behavior for macroscopically homogeneous and isotropic blends, thus it is valid for co-continuous systems:

E_b_^1/5^ = E_1_^1/5^φ_1_ + E_2_^1/5^φ_2_ (4)

**Figure 8:**
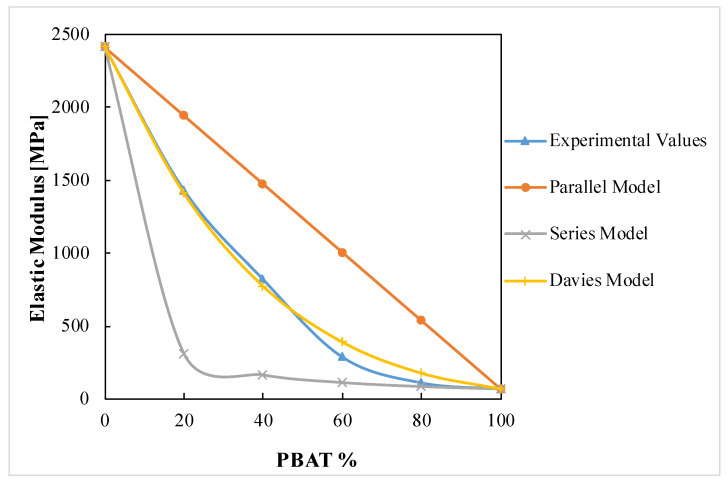
Elastic modulus in function of the blends’ composition.

The experimental result of the modulus and the theoretical predictions of the above-reported equations are given in Figure 8.

From Figure 8, it can be seen that the experimental values of the elastic modulus are between the highest and lowest limits of the parallel and series models. However, the model that fits best the experimental values is the Davies ones. Particularly, at 40%, there is a change in slope of the experimental values’ curve that intersects the Davies Equation in a composition between 40% and 60% of PBAT, as a further confirmation of the possible formation of a co-continuous phase in this range. Moreover, the elastic modulus of the blends with a PBAT content of 20% is the closest to the parallel model, a sign of the highest level of interactions of the two polymers in this composition, as revealed by the rheological and morphological analysis.

Few works in literature deal with the application of literature models to the mechanical properties of PLA/PBAT blends. Deng et al. [29] also found a similar trend with the experimental values of the elastic modulus comprised between the series and the parallel model, while Gigante et al. [36] found that the experimental values, up to a PBAT content of 25%, followed the Parallel model.

**Figure 9:**
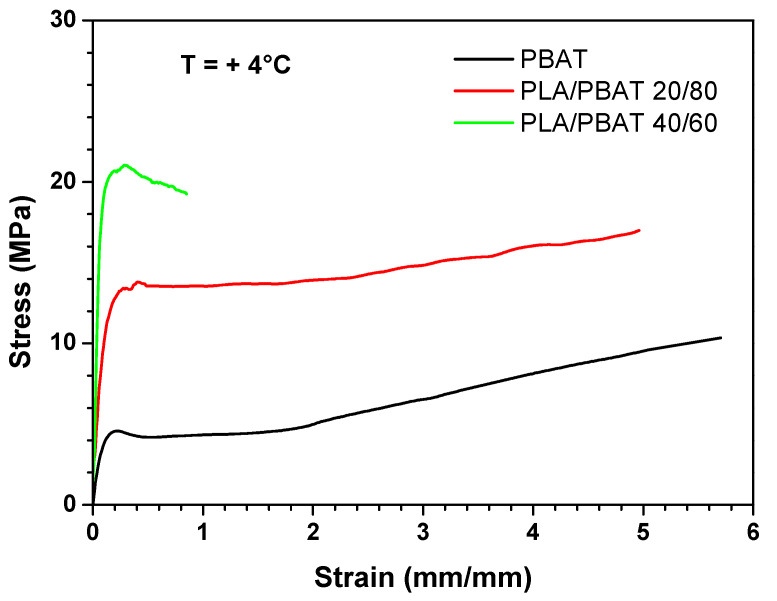
Stress–strain curves at + 4 °C for PBAT, PLA/PBAT 20/80 and PLA/PBAT 40/60 films.

**Figure 10:**
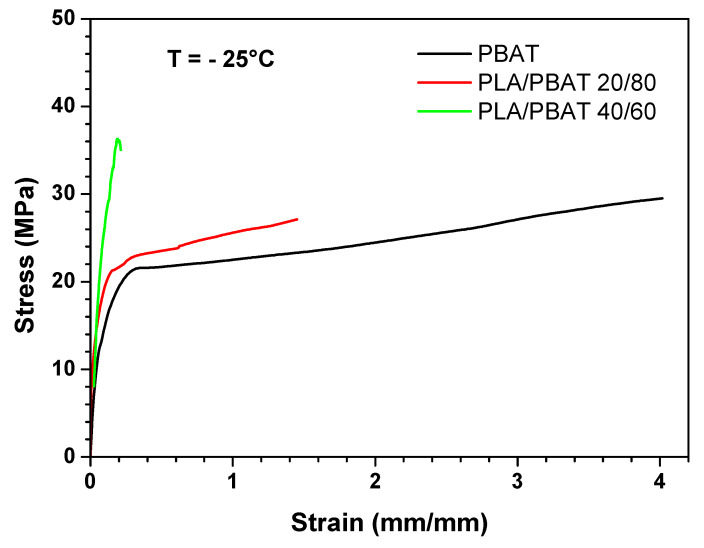
Stress–strain curves at −25 °C for PBAT, PLA/PBAT 20/80 and PLA/PBAT 40/60 films.

**Figure 11:**
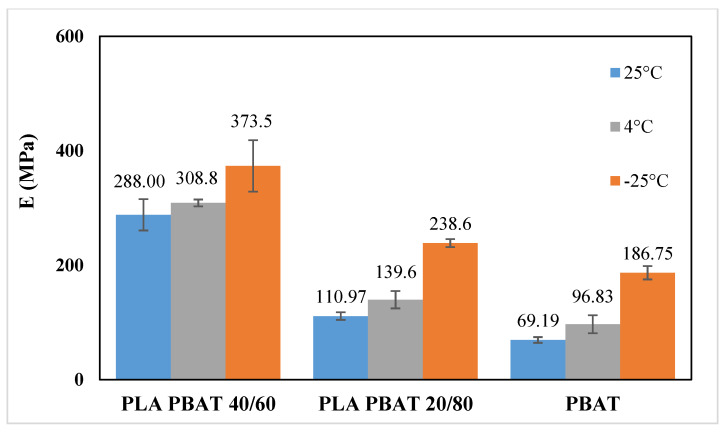
Comparison of elastic modulus at different temperatures.

**Figure 12:**
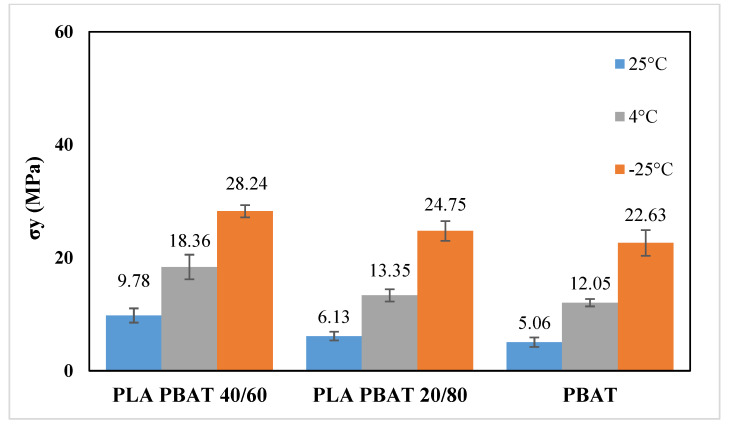
Comparison of yield at stress at different temperatures.

**Figure 13:**
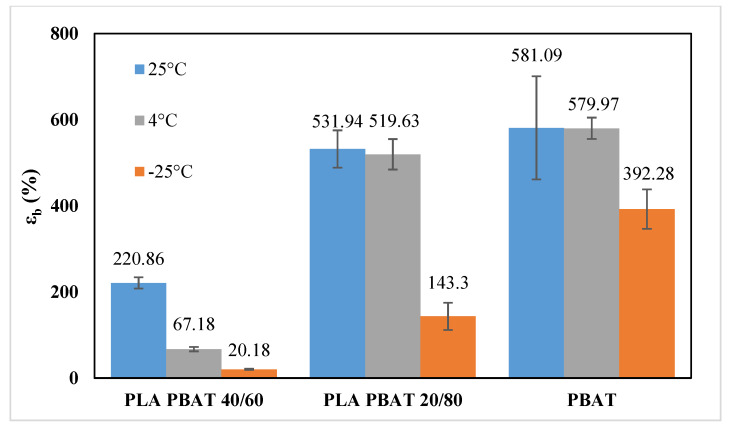
Comparison of the elongation at break at different temperatures.

From the analysis of the mechanical properties at ambient temperature, it is evident that the most ductile blends are those in which PBAT is the matrix phase (i.e., PLA/PBAT 40/60, PLA/PBAT 20/80 and PBAT). Therefore, the mechanical behavior of these blends was determined also at 4 °C, which is the typical fridge temperature, and at −25 °C, which is the typical temperature of the industrial freezer, in order to test their suitability as chilled and frozen food packaging materials. Figure 9 and Figure 10 show the stress–strain curves obtained at 4 °C and −25 °C, respectively, whereas Figure 11, Figure 12 and Figure 13 compare respectively the elastic modulus, the stress at yield, and the elongation at break for the PLA/PBAT 40/60, PLA/PBAT 20/80 and PBAT films at the three test temperatures.

From these graphs it is clear that the decrease in temperature caused, for all the tested blends, an increase in both elastic modulus and yield stress, and a decrease in the elongation at break. These results are expected, because the decrease in temperature reduces the polymer chain flexibility, and increases the stiffness of the material. However, this variation had a different extent in the function of the blend’s composition. In fact, the increase of the elastic modulus was more accentuated as the content of PBAT in the blend increased, varying from the 30% for PLA/PBAT 40/60 to 170% for pure PBAT (at −25 °C). This can be explained by the fact that, since the glass transition temperature of PBAT is close to −25 °C, the Elastic modulus has a higher dependence upon the temperature compared to PLA, whose elastic modulus in this temperature range (−25 °C to 25 °C) is almost constant. As regards the ductility, the effect of the composition was the opposite compared to the elastic modulus. In fact, the decrease in the elongation at break was more evident as the content of PLA increased, ranging from the 90% PLA/PBAT 40/60 to 30% for pure PBAT (at −25 °C). Particularly, the blend with a PLA content equal to 40%, at −25 °C turned its failure mode from ductile to brittle. This could be due the fact that, at all of the tested temperatures, PLA is in the glassy state, and instead PBAT is the rubbery state, so its ductility is less sensitive than PLA to the temperature decrease. In conclusion, both PLA/PBAT 40/60 and 20/80 could be good candidates for chilled food applications, showing at 4 °C a good compromise between stiffness and ductility. At −25 °C, the failure mode of PLA/PBAT 60/40 changed from ductile at ambient temperatures to brittle, while the PLA/PBAT 20/80 kept its ductile failure mode, revealing that it is the best candidate for frozen food applications.

## 4. Conclusions

In this study, blown films of PLA and PBAT in a wide range of compositions (100/0, 80/20, 60/40, 40/60 20/80 0/100 by weight) were produced using a lab-scale film blowing plant, and were characterized in terms of microstructure and functional properties in order to determine their suitability as food packaging materials for room and low temperature (chilling and freezing) storage conditions.

The two polymers, although immiscible in all the compositions tested, proved to have some interactions, as inferred by infrared spectroscopy. This causes that the microstructure of the blend films depends strongly on their composition. In particular, the presence of PBAT raises the crystallinity degree of PLA (from 8% of pure PLA, to 22% of the PLA/PBAT 20/80 blend), while the presence of PLA had the opposite effect on PBAT (from 15% of pure PBAT to 2% of the PLA/PBAT 80/20 blend). In terms of performances, the barrier properties to oxygen and water vapor, while it decreased with the increase of PBAT content according to the Robeson equation, remained in the same order of magnitude for all the compositions; P O_2_ varied from 33.4 to 84 (cm^3^·mm)/(m^2^·d·bar) and P H_2_O from 1.3 to 3.1 (g·mm)/(d·m^2^). Moreover, the ductility increases and the stiffness decreases, according to the Davies model, as the PBAT content in the blends becomes progressively higher, at all test temperatures. However, at fixed blend composition, the ductility decreased, and the stiffness increased the lower is the temperature.

On this basis, among the considered blown films, the following two blends were selected as best candidates as packaging materials for food requiring low storage temperatures, thanks to a balance of mechanical properties in the typical range of conventional polymer systems used for this kind of applications. In particular, the blend with a PBAT content of 60% proved to be adequate for chilled foods: in fact, at 4 °C (which is the typical fridge temperature) it had an elastic modulus equal to 308 MPa, and elongation at break of 67%. Instead, the blend with a PBAT content of 80% was adequate for frozen foods: in fact, at −25 °C (which is the temperature of an industrial freezer) it had an elastic modulus of 238 MPa and elongation at break equal to 143%.

## Figures and Tables

**Table 1:** Differential Scanning Calorimetry (DSC) results of the 1st Heating scan of the films.

I Heating											
	T_g_ PBAT [°C]	T_g_ PLA [°C]	T_cc_ [°C]	ΔH_cc_ [J/g]	T_m1_ PBAT [°C]	T_m2_ PBAT [°C]	ΔH_m_ PBAT [J/g]	T_m_ PLA [°C]	ΔH_m_ PLA [J/g]	X_c_ PBAT [%]	X_c_ PLA [%]
PLA	/	63.5	97.6	35.3	/	/	/	170.3	36.6	/	1.5
PLA PBAT 80 20	−33.1	62.1	97.2	21.7	/	/	/	168.6	27.9	/	8.3
PLA PBAT 60 40	−33.9	61.3	96.0	14.9	47.5	118.0	0.8	169.2	22.8	1.7	14.1
PLA PBAT 40 60	−34.1	61.5	96.4	8.0	48.2	111.8	2.3	168.8	15.3	3.3	19.4
PLA PBAT 20 80	−35.1	63.2	95.5	1.7	48.0	112.6	7.9	167.2	6.0	8.7	22.7
PBAT	−35.4	/	/	/	48.8	110.6	17.2	/	/	15.1	/

**Table 2:** Oxygen and water vapor permeability coefficients of the films.

	P O_2_ [cm^3^·mm/m^2^·d·bar]	P H_2_O [g·mm/(d·m^2^)]
PLA	33.4	1.3
PLA PBAT 80 20	40.9	1.4
PLA PBAT 60 40	50.3	2.8
PLA PBAT 40 60	61.9	2.9
PLA PBAT 20 80	71.8	3.0
PBAT	84.0	3.1

**Table 3:** Tensile properties of the films at 25 °C.

Mechanical Properties at Ambient Temperature			
Blend	E (MPa)	εb (%)	σy (MPa)
PLA	2411.0 ± 164.3	6.7 ± 1.2	45.3 ± 5.4
PLA PBAT 80 20	1432.1 ± 119.7	15.4 ± 3.8	39.1 ± 2.5
PLA PBAT 60 40	819.6 ± 73.1	183.0 ± 20.1	22.1 ± 2.3
PLA PBAT 40 60	288.0 ± 27.4	220.8 ± 12.9	9.7 ± 1.2
PLA PBAT 20 80	110.9 ± 6.6	531.9 ± 43.2	6.1 ± 0.7
PBAT	69.1 ± 5.0	581.1 ± 119.9	5.1 ± 0.8
